# Incidence Rates of Clinically Significant Tinnitus: 10-Year Trend From a Cohort Study in England

**DOI:** 10.1097/AUD.0000000000000121

**Published:** 2015-04-27

**Authors:** Carlos Martinez, Christopher Wallenhorst, Don McFerran, Deborah A. Hall

**Affiliations:** 1 Institute for Epidemiology, Statistics and Informatics GmbH, Frankfurt, Germany; 2 Colchester Hospital University NHS Foundation Trust, Colchester, Essex, United Kingdom; 3 National Institute for Health Research (NIHR) Nottingham Hearing Biomedical Research Unit, University of Nottingham, Nottingham, United Kingdom; and 4 Otology and Hearing Group, Division of Clinical Neuroscience, School of Medicine, University of Nottingham, Nottingham, United Kingdom.

**Keywords:** Clinically significant tinnitus, Incidence rates, Time trend

## Abstract

**Objective:**

To investigate the incidence of tinnitus that burdens the health service in England.

**Design:**

This was an observational study of 4.7 million residents of England under 85 years of age who were at risk for developing clinically significant tinnitus (sigT). SigT was defined by a discharge from hospital with a primary diagnosis of tinnitus, or a primary care recording of tinnitus with subsequent related medical follow-up within 28 days. The database used was the Clinical Practice Research Datalink and individual records were linked to additional data from the Hospital Episode Statistics. The observational period was from January 1, 2002 to December 31, 2011. Age-, gender-, and calendar year-specific incidence rates for and cumulative incidences of sigT were estimated and a projection of new cases of sigT between 2012 and 2021 was performed.

**Results:**

There were 14,303 incident cases of sigT identified among 26.5 million person-years of observation. The incidence rate was 5.4 new cases of sigT per 10,000 person-years (95% confidence interval: 5.3 to 5.5). The incidence rate did not depend on gender but increased with age, peaking at 11.4 per 10,000 in the age group 60 to 69 years. The annual incidence rate of sigT increased from 4.5 per 10,000 person-years in 2002 to 6.6 per 10,000 person-years in 2011. The 10-year cumulative incidence of sigT was 58.4 cases (95% confidence interval: 57.4 to 59.4) per 10,000 residents. Nearly 324,000 new cases of sigT are expected to occur in England between 2012 and 2021.

**Conclusions:**

Tinnitus presents a burden to the health care system that has been rising in recent years. Population-based studies provide crucial underpinning evidence; highlighting the need for further research to address issues around effective diagnosis and clinical management of this heterogeneous condition.

## Abstract 2

This study used national clinical data from the general English population to estimate the incidence of “clinically significant” tinnitus (sigT), defined as tinnitus of sufficient concern to warrant seeking medical medical assistance. The annual incidence rate of sigT was 6.6 per 10,000 person-years, corresponding to 31,000 new cases in England in 2012. The incidence rate increased with age from 0.3 for those under 10 years to 12.7 per 10,000 person-years in those ages 60-69. Nearly 324,000 new cases of sigT are predicted to occur between 2012 and 2021. These findings demonstrate the considerable burden of sigT at a population level.

Supplemental Digital Content is available in the text.

## INTRODUCTION

Tinnitus remains one of the most common auditory symptoms. Depending on the definition of tinnitus and the criteria applied, prevalence rates in adult populations vary from 8.2 to 20.0% (Fabijanska et al. 1999; Nondahl et al. 2002), rising to 17.9 to 30.3% in those over 50 years of age (Sindhusake et al. 2003; Nondahl et al. 2004; Shargorodsky et al. 2010).

Published UK prevalence rates are in broad agreement with these estimates. The most comprehensive UK survey undertaken was by the National Study of Hearing dating from the 1980s (Davis 1989) and this yielded a prevalence estimate of tinnitus of 10.2% in the total adult population, rising to 14.2% in those over 50 years of age. Recent data collected between 2006 and 2010 as part of the UK Biobank resource showed a 16.9% prevalence for adults ages 40 to 69 years (Dawes et al. 2014). In both studies, tinnitus was defined as self-reported prolonged spontaneous tinnitus lasting for more than 5 minutes at a time.

While prevalence estimates for tinnitus provide a snapshot of the potential population burden of this condition, incidence studies consider *new* cases and so can assess the risk of developing tinnitus over a period of time. Two prospective cohort studies, Beaver Dam, Wisconsin, USA (Nondahl et al. 2002, 2010) and Blue Mountains Hearing Study, Sydney, Australia (Gopinath et al. 2010), have so far assessed the long-term incidence of tinnitus in the general population. In the Beaver Dam cohort, 2922 older adults showed a 5-year cumulative incidence of tinnitus of 5.7%, and a 10-year cumulative incidence of 12.7% (Nondahl et al. 2002, 2010). In the Blue Mountains cohort, the 5-year cumulative incidence of tinnitus was 18.0%, calculated from 612 older adults (Gopinath et al. 2010).

While previous research goes some way toward addressing the incidence of self-reported tinnitus and its perceived severity in the general population, it does not provide insight into the impact and burden of chronic tinnitus from a health care perspective. In this study, we have used national clinical data to examine temporal trends in the incidence of what we have termed “clinically significant” tinnitus, a tinnitus that is judged to be of sufficient concern to warrant seeking medical assistance at both primary and secondary care levels, in the general English population over a 10-year period.

## MATERIALS AND METHODS

### Data Sources

This study used openly available anonymized patient-based primary care data from the United Kingdom Clinical Practice Research Datalink (CPRD) of which a subset has been linked to the “Admitted hospital care” data of Hospital Episode Statistics (HES) by a trusted third party (Patient-based Linkage, CPRD 2014). The United Kingdom comprises four countries, namely England, Scotland, Wales, and Northern Ireland. Although some administration is still centralized, various government functions have been devolved to the individual countries. Health care is one of those devolved services and the HES figures are those pertaining to England, not the other three countries. As of November 2013, the CPRD data linked to HES data comprised 5.76 million active patients from 364 primary care practices throughout England before January 1, 2012.

Primary care data contain demographics, medical diagnoses and symptoms, prescriptions issued by general practitioners (GPs), test results, and referrals to secondary care (UK CPRD 2014). Diagnoses and symptoms are recorded using Read medical codes, a coded thesaurus of clinical terms used since 1985. Read Codes are the basic means by which clinicians record patient findings and procedures in health and social care information technology (IT) systems across primary and secondary care in the UK (e.g., general practice surgeries and pathology reporting of results). Read Codes are still widely used in the primary care sector. Prescriptions are recorded with Gemscript codes, a set of data based on the UK National Health Service (NHS) dictionary of medicine and devices. It describes the clinical, commercial and physical attributes of the medicines, medical devices, and appliances used in the health care industry. CPRD also includes socioeconomic information provided by quintiles of the Townsend deprivation index, a measure of material deprivation calculated using census data and linked to area of residence (Townsend et al. 1988). HES contains details of all hospital admissions, inpatient procedures and interventions, discharge diagnoses, and outpatient appointments at NHS hospitals in England (Health & Social Care Information Centre 2014). Discharge diagnoses are recorded with ICD-10 codes. OPCS-4 codes have been used for the classification and coding of in-hospital interventions and procedures and are part of HES data.

Age-specific mid-year population estimates for the years 2002–2011 and age-specific mid-year projections for the years 2012–2021 for England were provided from the Office for National Statistics (Office for National Statistics 2011a, 2011b).

### Study Population

The study population consisted of all individuals below 85 years in the CPRD-HES link registered with a general practitioner during the study period of January 1, 2002 to December 31, 2011, and with at least 365 days of history in the CPRD-HES link. This age restriction was set, as it is thought that detection and recording of tinnitus may be less complete in the elderly for various reasons.

**Fig. 1.**
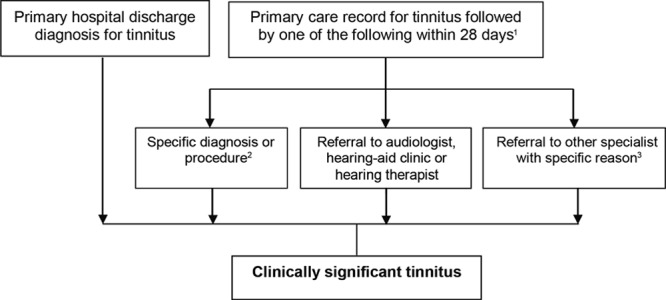
Algorithm for detection of clinically significant tinnitus (sigT). Superscript numbers denote: ^1^Comprises a diagnosis for tinnitus, see Supplemental Digital Content 1 (http://links.lww.com/EANDH/A172) for specific code set. ^2^Comprises a diagnosis or procedure on the external, middle or inner ear or acoustic nerve, including hearing loss, Ménière’s disease, vestibular disorders, procedures to treat otosclerosis, and fitting of a hearing aid or implantation of a hearing device, see Supplemental Digital Contents 2, 3, and 4 (http://links.lww.com/EANDH/A172) for specific code sets. ^3^Consists of referrals to other specialist (otorhinolaryngology, neurology, radiology, psychiatry, psychology or counseling) with a specific referral reason including a hearing test, hearing-related problems, tinnitus, a diagnosis involving otosclerosis, Ménière’s disease, other vestibular disorders or a related procedure on the external, middle or inner ear or acoustic nerve, see Supplemental Digital Content 5 (http://links.lww.com/EANDH/A172) for specific code sets.

The study outcome was a first recording of clinically significant tinnitus (sigT) defined by a case ascertainment algorithm (Fig. 1) when one of the following situations existed:

1. A patient was discharged from hospital with a primary discharge diagnosis of tinnitus defined with ICD-10 code H931, and

2. A patient had a primary care recording of tinnitus and one of the following conditions were recorded or qualifying clinical events were met within 28 days after the tinnitus recording: (i) a specific diagnosis or procedure, (ii) a referral to an audiologist, a hearing-aid clinic or a hearing therapist, or (iii) a referral to other specialist with a specific referral reason.

The date of onset of sigT (index day) for in-hospital cases was the discharge day and for ambulatory cases the most recent recording of tinnitus preceding the qualifying case definition of a sigT event.

The observational period, that is, the time in which a patient from the study population (patients with a CPRD-HES link) was at-risk for developing sigT, started on January 1, 2002, at the individual’s completion of at least 1-year with the general practice or 1-year after the practice started to contribute valid data to the CPRD (up-to-standard date), whichever was later, and it ended when the patient left the practice, became 85 years, died, experienced a sigT, or the study period ended on December 31, 2011, whichever occurred first. Patients with a sigT before 2002 were excluded from all analyses as only new cases were evaluated.

### Data Analysis Methods

Crude incidence rates (IRs) of sigT were calculated from the number of new cases of sigT divided by the total person-years at risk of the study population. The total person-years at risk of the study population for IR estimations was calculated as follows: We counted the number of days between the beginning and end of each patient’s observational period, added up the number of days of all patients in the study population and divided the result by 365.25 days per year to get person-years instead of person-days. IRs were stratified by age, gender, and calendar year. A variance-weighted linear regression model was used to fit a straight line to the specific annual IRs of sigT from 2002 to 2011 and to estimate the slope of the regression line. A two-sided *t*-test was conducted to test whether the slope was different from zero.

Cumulative incidence estimates of sigT were derived using Kaplan–Meier estimates.

The estimated number of cases of sigT in all England between 2002 and 2011 were calculated from age-specific IRs of sigT and respective calendar year-specific population estimates in each 10-year age group (Office for National Statistics 2011a). Numbers of expected cases of sigT in the future were calculated by multiplying average age-specific IRs of sigT based on the years 2009–2011 with the age-specific projected English population (Office for National Statistics 2011b) for each year between 2012 and 2021 in each 10-year age group. Upper and lower bounds for the total number of expected cases of sigT in the future were calculated using the lower and upper bounds of the 95% confidence intervals (CIs) of the age-specific IRs in the years 2009–2011. For the estimate over all age groups, a range from the sum of lower limits of age-specific 95% CI to the sum of upper limits was provided.

Sensitivity analyses on the time window between tinnitus recording and additional referral/procedure to define sigT were performed by calculating the overall IR of sigT using a time window of 21 and of 35 days instead of 28 days.

All statistical procedures were performed using the STATA MP Version 13.1 (StataCorp LP). The study protocol was approved by the Independent Scientific Advisory Committee for CPRD research.

## RESULTS

**Fig. 2.**
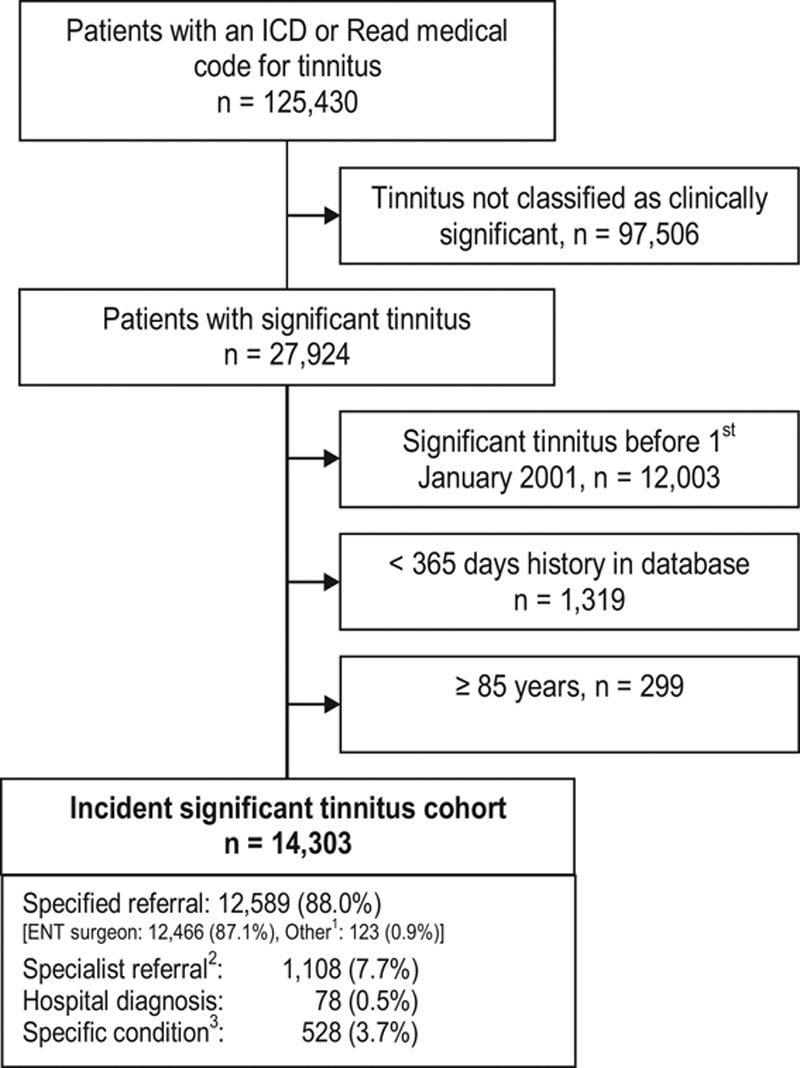
Ascertainment of patients with incident clinically significant tinnitus (sigT). ENT, ear, nose, and throat; Superscript numbers denote: ^1^Consists of referrals to other specialist (otorhinolaryngology, neurology, radiology, psychiatry, psychology, or counseling) with a specific referral reason including a hearing test, hearing-related problems, tinnitus, a diagnosis involving otosclerosis, Ménière’s disease, other vestibular disorders or a related procedure on the external, middle or inner ear or acoustic nerve, see Supplemental Digital Content 5 (http://links.lww.com/EANDH/A172) for specific code sets. ^2^Consists of referrals to audiologist, hearing aid clinic, or hearing therapist. ^3^Comprises a diagnosis or procedure on the external, middle or inner ear or acoustic nerve, including hearing loss, Ménière’s disease, vestibular disorders, procedures to treat otosclerosis, and fitting of a hearing aid or implantation of a hearing device, see Supplemental Digital Content 2, 3, and 4 (http://links.lww.com/EANDH/A172) for specific code sets.

The study population comprised 4,703,226 persons in England with mean age of 34.7 years 50.5% females and mean body mass index of 24.8 kg/m^2^ (Table 1). The study population contributed a total of 26.5 million person-years of observation between January 1, 2002 and December 31, 2011. In this population, we identified 125,430 patients with any tinnitus. After applying the definition of sigT, 14,303 patients with a first-time diagnosis of sigT were found during the complete observational period (Fig. 2). Of those, 12,589 (88.0%) were identified because of a referral to a prespecified specialist, with nearly 99% of those being to an otorhinolaryngologist. The remaining patients with sigT were identified from a hospital diagnosis (0.5%), specific conditions (3.7%), and referrals to other relevant hearing professionals, which included audiologists, hearing aid clinics, and hearing therapists (7.7%).

**Table 1.**
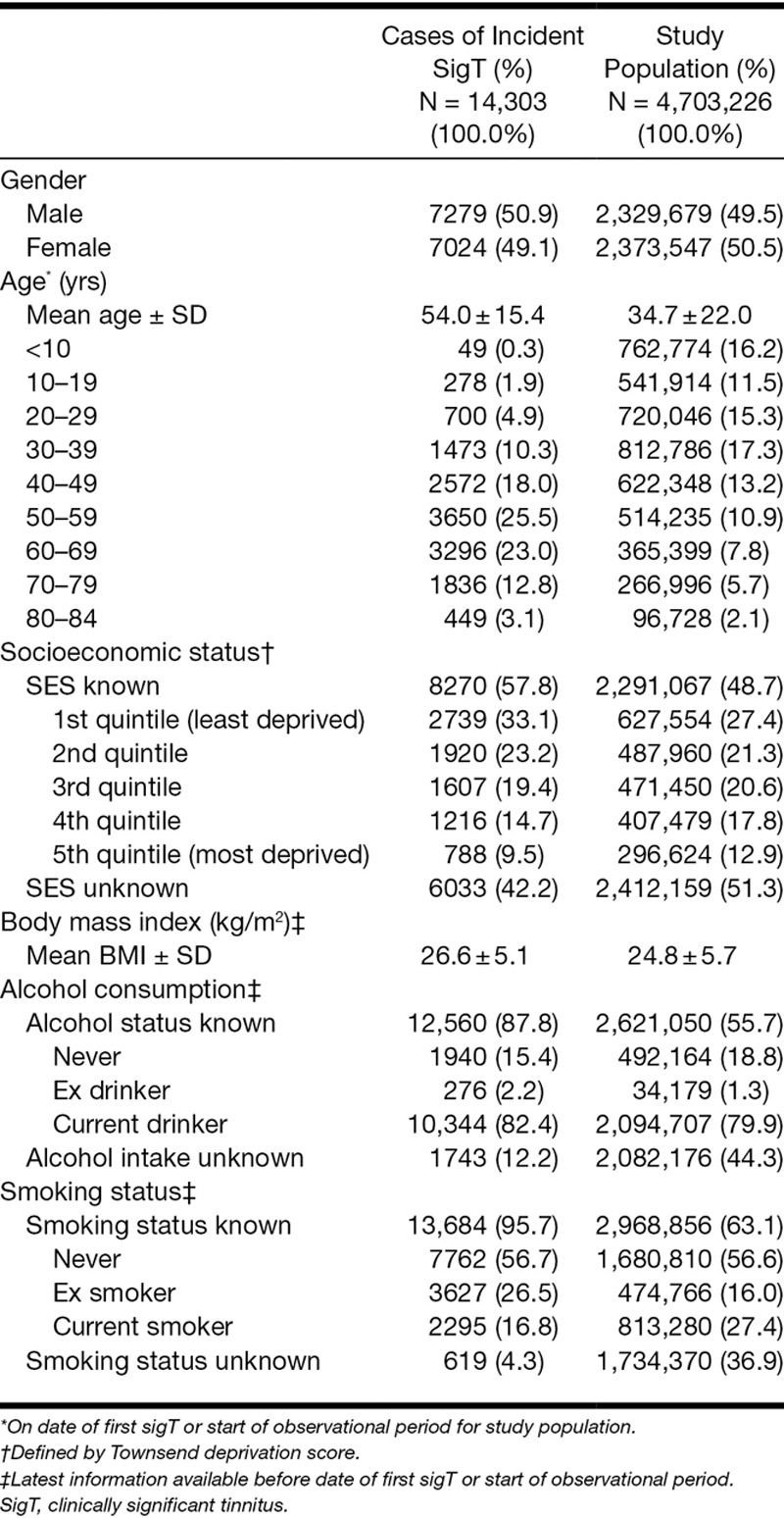
Characteristics of the study population and of patients with incident clinically significant tinnitus (sigT)

The mean age of the 14,303 incident cases of sigT was 54.0 years (SD = 15.4). SigT was recorded in all age groups, but most were ages 50 to 69 (48.5%). The age distribution and other descriptive characteristics of the incident cases are reported in Table 1. There were broadly equivalent numbers of males and females. A larger proportion had a higher socioeconomic status (33.1% in least deprived quintile versus 9.5% in most deprived quintile) and had never smoked (56.7%). The mean body mass index was 26.6 kg/m^2^.

**Fig. 3.**
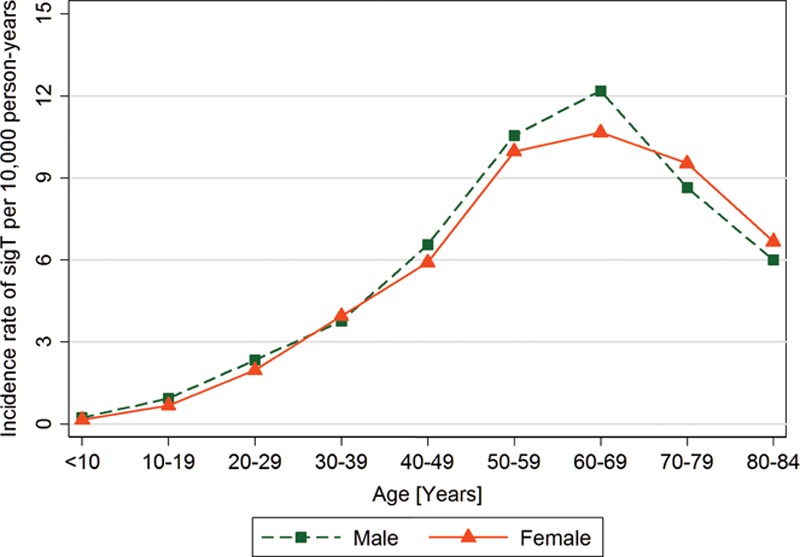
Gender- and age-specific incidence rates of clinically significant tinnitus (sigT).

The overall IR of sigT, that is, the number of new cases of sigT per total person-time at risk, calculated from the sum of each individual’s time at risk, across all ages during the 10-year observational period, was 5.4 new cases of sigT per 10,000 person-years (95% CI: 5.3 to 5.5). In a sensitivity analysis, we examined the effect of the chosen 28-day time window defining sigT on the previously observed result. For inclusion into the present study, sigT was defined as a tinnitus recording with additional referral/procedure within 28 days or a hospital primary discharge diagnosis of tinnitus. To address this question, we specified two other time windows (21 and 35 days), and compared the results to those from the analysis with a 28-day time window. The shorter time window of 21 days resulted in an overall IR of 5.4 (95% CI: 5.3 to 5.5) per 10,000 person-years, and the longer time window of 35 days resulted in an overall IR of 5.5 (95% CI: 5.4 to 5.6). Thus, the results were almost unchanged and not sensitive to the applied time window. Figure 3 illustrates the pattern of gender- and age-specific IRs of sigT. We observed incident sigT even in children under 10 years indicating that one in 50,000 children under 10 met our criteria for incident sigT every year, based on the overall IR estimate for sigT in the age group of patients under 10 years. Overall, the IR increased steadily with age up to 11.4 per 10,000 person-years in people ages 60 to 69, and it declined thereafter. With respect to gender, men and women had similar IRs in all age groups (Fig. 3, Supplemental Digital Content 6, http://links.lww.com/EANDH/A172).

**Fig. 4.**
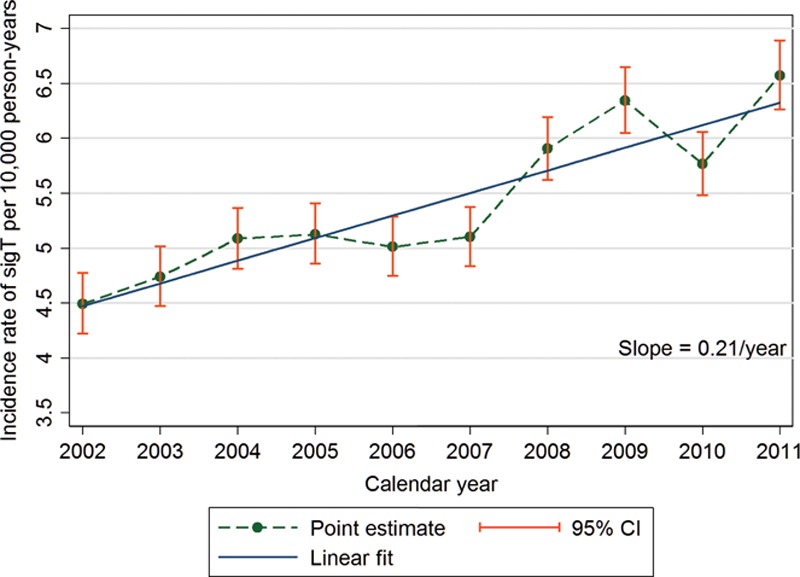
Incidence rates of clinically significant tinnitus (sigT) by calendar year with confidence intervals (CI) and fitted linear trend.

Figure 4 illustrates the IR over the study period, stratified by calendar year. The annual IR of sigT increased from 4.5 per 10,000 person-years in 2002 to 6.6 per 10,000 person-years in 2011 (Fig. 4, Supplemental Digital Content 7, http://links.lww.com/EANDH/A172). The estimated increase in cases of sigT per 10,000 person-years was 0.21 (95% CI: 0.13 to 0.28) per calendar year (Fig. 4). This trend for an increasing incidence over time was statistically significant (*p* < 0.001) despite a dip in 2010.

The 10-year cumulative incidence of sigT across all ages, that is, the proportion of individuals who experienced (first) sigT during a defined time interval, was 58.4 per 10,000 (95% CI: 57.4 to 59.4), peaking at 112.2 per 10,000 (95% CI: 108.6 to 115.9) in ages 50 to 59. Thus, one person out of every 171 was likely to have experienced sigT in the 10-year period of the study (Table 2).

**Table 2.**
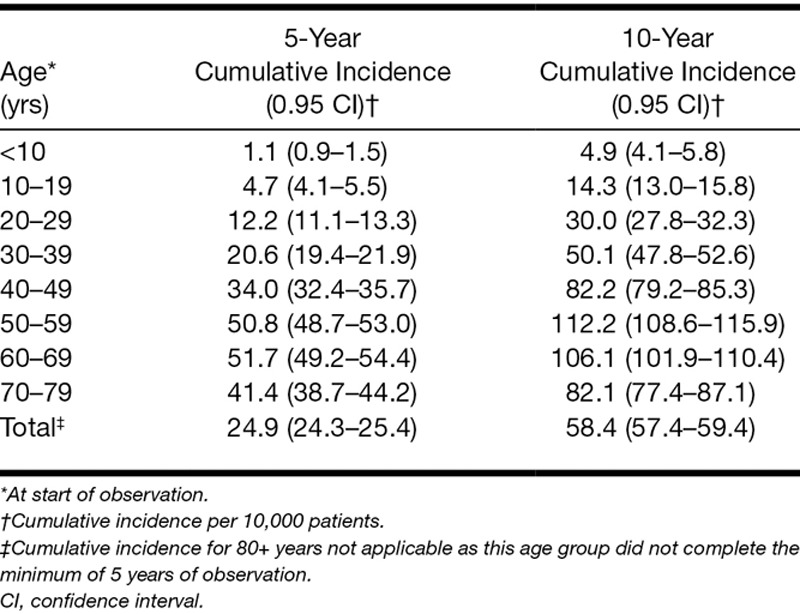
Kaplan–Meier estimates for 5-year and 10-year cumulative incidence of clinically significant tinnitus after start of observation, stratified by age at start of observation

The total number of incident cases of sigT in England over the 10 years (2002–2011) is estimated at approximately 258,000 (Table 3). The figures obtained in this study can be used to estimate the public health burden of tinnitus in the near future; based on the average IR of sigT in the last 3 years of the study (2009–2011), around 324,000 (range from the sum of lower limits of age-specific 95% CI to the sum of upper limits: ca. 300,000 to 350,000) new cases of sigT are expected to occur in England between January 2012 and December 2021 (Table 4).

**Table 3.**
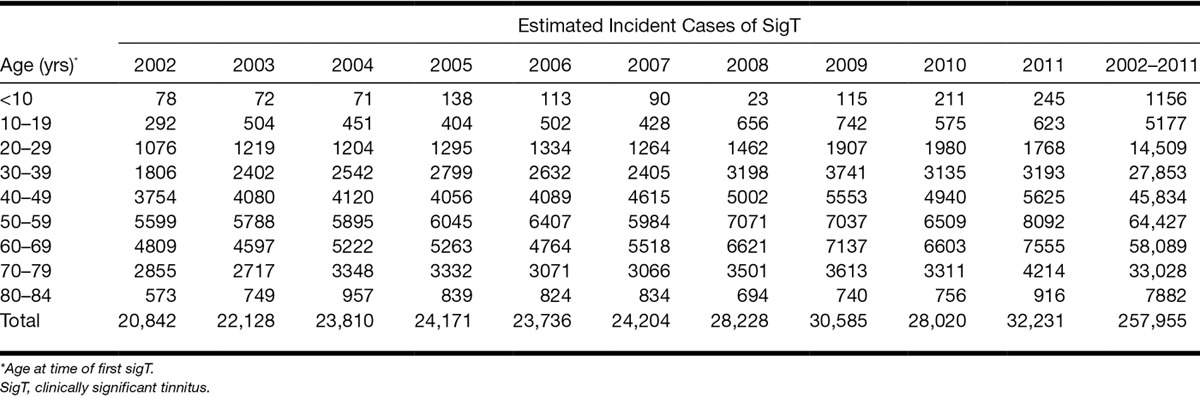
Estimated number of incident clinically significant tinnitus cases in England, between January 2002 and December 2011

**Table 4.**
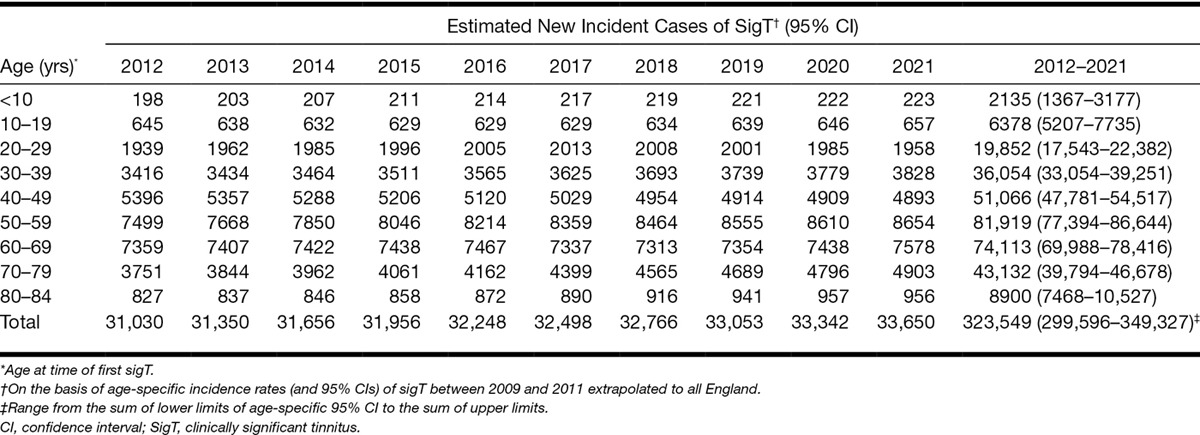
Predicted number of new clinically significant tinnitus cases in England, between January 2012 and December 2021 assuming a constant IR based on the observed age-specific IR over years 2009–2011

## DISCUSSION

To our knowledge, this is the first study in a large population sample on the clinical burden of patients with first-time experience of clinically sigT that leads the individual to seek additional resources from within the NHS health care system. One of the major strengths of this national study is that it represents a very large cohort with more than 26 million person-years of observation. These reported data therefore provide an important perspective on the large-scale burden of tinnitus on a population scale. This has allowed us to accurately describe the epidemiology of sigT in much more detail than hitherto and in the broad population of the whole country rather than just a specific region (Nondahl et al. 2002, 2010) or age cohort (Dawes et al. 2014).

Our main finding, as confirmed by the significant non-zero slope of the fitted regression line, indicates a general tendency for an annual rise in IR of 2.1 cases per 10,000 person-years between 2002 and 2011. This pattern may reflect increased prevalence, higher health expectations leading to increased detection, or increased awareness also leading to increased detection (Nondahl et al. 2012). Perhaps the latter two explanations are the more likely given recent evidence that hearing loss (the major risk factor for tinnitus) may be declining among older adults compared with earlier generations of the same age (e.g., Hoffman et al. 2010).

Higher health expectations among more recent generations of UK adults are a likely contributor to the growth in the number of instances of medical help-seeking for tinnitus (Ham et al. 2012). The long period of postwar economic growth has benefited those “baby boomers” born in the 1940s and early 1950s. This generation has now reached retirement with greater wealth than at any time in history, and is at the age of most intensive use of NHS services for tinnitus (Fig. 3). In support of this view, our findings show that more cases of sigT were seen among patients with a higher economic status, that is, those who are most likely to expect (or insist upon) onward referral (Ham et al. 2012).

Improved public and patient awareness of symptoms is provided by the growth in internet use. For example, a recent “Google” search identified over 11 million web pages devoted to tinnitus information, treatment options, treatment clinics, self-help strategies, and discussion forums (Fackrell et al. 2012). Much easier access to information through the internet has started to change the relationship between patients and professionals. Patients are less likely to consult their GP as passive recipients and are more likely to have sought information themselves before the GP appointment and to be more demanding about access to onward services (Realpe & Wallace 2010).

### Comparison With Previous Studies

The IRs in our study are not directly comparable with previous incidence reports. First the definition of the condition is not the same, even across already published studies. For example, the lower incidence estimates in the Beaver Dam study compared with the Blue Mountains study are possibly attributable to the more stringent definition of tinnitus which the authors of the former work defined as having buzzing, ringing, or noise in the ears in the past year that was at least moderate in severity or that caused problems getting to sleep (Nondahl et al. 2002). Not only is our definition very different, but also we used confirmed diagnoses by a health care professional, rather than participant self-reports (Nondahl et al. 2002, 2004, 2010; Gopinath et al. 2010; Nondahl et al. 2012). Despite these differences, two important common themes emerge.

First, the age-specific IRs of sigT mirror the same age trends, notably the IR of sigT increasing steadily with age up to 60 to 69 years and then declining thereafter. For example, data from the UK National Study of Hearing reported that the prevalence of prolonged spontaneous tinnitus increases up to the age category 61 to 70 years (14%) and drops to 4% for people over 80 years of age (Davis 1989). Recent data from the UK Biobank also indicate a steadily growing prevalence with age across the 40 to 69 year old sample studied (McCormack et al. 2014). Cohort studies from other countries report the same age trend, even though the absolute prevalence may differ (Hoffman & Reed 2004). For example, in the Beaver Dam study, prevalence increased up to age 60 to 69, remained high between 70 and 79 years, and then declined for the oldest age group ≥80 years (Nondahl et al. 2002; see also Hoffman & Reed 2004).

Second, no difference between men and women was seen in the IRs of sigT. The published literature is rather mixed. For example, neither Shargorodsky et al. (2010) nor Sindhusake et al. (2003, 2004) reported gender differences, while Nondahl et al. (2010) reported a higher incidence for men. Data from the UK Biobank indicates men were more likely to experience tinnitus, but women were more likely to find their tinnitus bothersome (McCormack et al. 2014).

### Burden to the Health Care System

Tinnitus has remarkable heterogeneity across the population. Subjective tinnitus is a symptom that is associated with practically every known ear disorder and so if a patient complains of the condition, it is appropriate to examine the range of possible otological etiologies. On the basis of the incidence estimates reported in the present study, the yearly cost of tinnitus to the NHS in England could easily exceed £4.9 million, because this estimate alone is based on one GP appointment (£45; Personal Social Services Research Unit 2013) and one outpatient appointment (£108; NHS 2014) for each incident case in 2011. If any additional prescriptions, diagnostic assessments, procedures, or referrals are required, then this would of course markedly elevate the health economic costs. Evaluation of the socioeconomic burden of tinnitus is beyond the scope of the present study.

### Study Limitations

Several pieces of evidence lead us to conclude that the data reported here may actually underestimate the number of cases of tinnitus that drives people to seek help from health care professionals. For example, in a recent survey of GPs across England, data indicated that there may be as many as 0.75 million patient consultations every year in England alone where tinnitus is the presenting symptom (El-Shunnar et al. 2011). Clearly, while the GP may make a primary care recording of tinnitus, not all of these cases are referred for onward audiological or ear, nose, and throat investigation and therefore did not meet the criteria in our case ascertainment algorithm. Referral behavior is known to differ amongst GPs, but on average GPs in England typically refer only 37% of their tinnitus patients (El-Shunnar et al. 2011).

It is noted that the IR is based on the date of first recording of sigT defined as a recording of tinnitus in primary care and a subsequent relevant referral or specific procedure within 28 days or as the date of admission for those with a hospital primary discharge diagnosis of tinnitus, and it is not based on the date of first occurrence of tinnitus. It is assumed that patients will approach their GPs or receive their hospital primary discharge diagnosis soon after their tinnitus becomes significant.

While we do acknowledge that onward referral to specialist services is not always necessary (Department of Health 2009), previous studies have suggested that GPs may not be effectively triaging their patients perhaps through a general lack of knowledge about tinnitus or an apparent insensitivity to its effect on quality of life (Davis et al. 2012). More specifically, tinnitus in children has been regarded as an uncommon problem, rarely noted by pediatricians. Our study indicates that, for children under 10 years old, the 10-year cumulative incidence of sigT was 4.9 per 10,000. This low figure may reflect in part the difficulty of a reliable clinical diagnosis (Savastano 2007; Shetye & Kennedy 2010).

While we observed 258,000 retrospective incident cases of sigT across England over the past 10 years, we predict that about 324,000 new cases of sigT might occur in the next 10 years. Our estimate is based on the assumption that the future age specific IRs of sigT in the next 10 years will not differ from the average IRs seen between 2009 and 2011. The 324,000 predicted new cases will be an underestimation of the number of total sigT cases if the increase in the IR of sigT that was seen between 2002 and 2011 continues in the next 10 years (e.g., due to increase in tinnitus risks or better detection through a higher grade of diagnosis or referrals).

### Concluding Remarks

In the present population study, we refer to the reporting of tinnitus symptoms in England followed by assessment, diagnosis, and/or management by relevant health care professionals as clinically significant tinnitus. Tinnitus is more challenging than many other health conditions, because there is no objective measurement of the condition, no consensus regarding diagnostic assessment, and no standardization of the management pathway (Hoare & Hall 2011). Population-based studies provide crucial underpinning evidence; highlighting the need for further research to address these issues and to provide effective care that is tailored to the needs of each individual patient.
